# Justification of physical intimate partner violence among married men in East Africa evidence from the recent demographic and health survey (2015–2022): a multilevel analysis

**DOI:** 10.3389/fsoc.2025.1514917

**Published:** 2025-06-27

**Authors:** Kaleb Assegid Demissie, Demiss Mulatu Geberu, Getachew Teshale, Melak Jejaw, Misganaw Guadie Tiruneh, Tesfahun Zemene Tafere, Asebe Hagos, Lemlem Daniel Baffa

**Affiliations:** ^1^Department of Health Systems and Policy, Institute of Public Health, College of Medicine and Health Science, University of Gondar, Gondar, Ethiopia; ^2^Department of Human Nutrition, Institute of Public Health, College of Medicine and Health Science, University of Gondar, Gondar, Ethiopia

**Keywords:** justification of physical IPV, married men, East Africa, DHS, pooled

## Abstract

**Introduction:**

Millions of women and girls worldwide are impacted by physical intimate partner violence. While physical intimate partner violence (IPV) among women is largely associated with the justification of IPV, little is known about men's attitude toward physical IPV. The aim of our study was to examine the factors associated with the justification of physical IPV among men in East Africa.

**Method:**

The study used data from the male file (MR) of the most recent demographic and health survey, which was carried out in 10 East African countries. a weighted sample of 74,494 men who were either married or living with a partner as if married. Multilevel logistic regression models were used to examine the relationship between the independent variables and justification of physical IPV.

**Result:**

The pooled prevalence justification of physical intimate partner violence in 10 east African countries was 24.17% (95% CI: 19.45–28.90). The highest prevalence of justification of physical IPV was in Uganda (35.31, 95% CI: 34.09–36.53), and the lowest prevalence of justification of physical IPV was in Malawi (8.88, 95% CI: 8.03–9.73). The multilevel analysis shows that men's age, working status, respondents' educational level, number of wives, and household wealth status, sex of head of household, age of the household head, place of residence, as well as community level poverty, and community level education were factors associated with the justification of physical intimate partner violence.

**Conclusion:**

In East Africa, about 24% of men agreed that physical IPV is justified. Reducing the justification of physical IPV requires advancing men's educational standing, men's economic status, and increased media awareness, with a focus on rural men and promoting educational and awareness campaigns at community level is needed.

## Introduction

The United Nations defines violence against women as any act of gender-specific violence that will result in sexual, physical, or mental harm for women, including intimidation, arbitrary denial of freedom, and threats of such acts, whether the deed is done in public or privately (Fried, 2003). Physical intimate partner violence is a traumatic life incident that can harm the health and wellbeing of a women (Plichta, 2004). In 2018, the World Health Organization (WHO) conducted an analysis of prevalence data from 2000 to 2018 in 161 countries and areas on behalf of the UN Interagency Working Group on Violence Against Women. The findings indicated that ~30% of women globally have experienced physical and/or sexual violence, either from an intimate partner, non-partner sexual violence, or both (World Health Organization, 2021). Social beliefs that encourage violence are the driving force behind these acts of violence, and these attitudes are connected to gender roles. Some men think that using violence helps to control their relationships and validate their masculinity (Seloilwe and Thupayagale-Tshweneagae, 2015). A study conducted in southern part Africa found that 25% of men justify wife-beating (Tsawe and Mhele, 2022), also, the notion that “wife-beating” is appropriate was least prevalent among both women and men in Central and Eastern Europe, Latin America, and the Caribbean, and most prevalent in Africa and South Asia (Tran et al., 2016). Men who are in favor of spousal abuse are also more prone to employ violence against their partners, children, or any other family members (Lansford et al., 2020). However, not much research has been done in eastern Africa to determine the prevalence and what factors are most important in affecting men's attitudes toward physical IPV.

More than 25% of women between the ages of 15 and 49 years who are in relationships report having experienced physical or sexual abuse at least once in their lifetime (World Health Organization, 2024). In low and middle-income countries, the prevalence of intimate partner violence was 37.2%, in central sub-Saharan Africa (44%), and in eastern sub-Saharan Africa (38%) (Ma et al., 2023; Sardinha et al., 2022). Among reproductive-age women in East African the prevalence of intimate partner violence was 43.72% (Tessema et al., 2023). Intimate partners are responsible between 38 and 50% of all murders of women; IPV also affects families, communities, and societies on a social and economic level (World Health Organization, 2024; UNODC, 2018). Globally speaking, women's conditions were generally the poorest in sub-Saharan Africa, highest incidence was seen in central and eastern Africa (The East African, 2022).

Despite this several initiatives and agendas have been implemented like, Goal 5 of the Sustainable Development Agenda clearly states that gender equality is a global priority (United Nations, 2015). Gender-related health initiatives, microfinance, women's empowerment have been implemented as interventions (Huis et al., 2017; Mandal et al., 2017). Also, using the multi-strategy, Relationship skills strengthened, Empowerment of women, Services ensured, Poverty reduced and Transformed attitudes and beliefs (R.E.S.P.E.C.T.) framework can significantly reduce the likelihood that women will experience intimate partner violence (Ward and Harlow, 2021). Different laws and regulations have been passed to stop violence against women, The Maputo Protocol of the East African Community (EAC) establishes strict guidelines for governmental accountability with relation to gender-based violence against women (GVAW) requiring the prevention, prosecution, and eradication of all forms of GVAW (EAC, 2021). Each country's passing their own laws; for example, Kenya (Act No. 2 of 2015) and Rwanda (No. 59/2008 of 10/09/2008) are currently standing out as the nation's having GVAW legislative and policy frameworks that are more extensive (UNHCR Rwanda, 2008; Domestic Violence Act of Kenya, 2015). Zimbabwe passed the Domestic Violence Act in 2007, while Zambia enacted the Anti-Gender-Based Violence Act No.1 in 2011 (Makahamadze et al., 2012; Advocats Sans Frontieres, 2017). But several of these laws and regulations had flaws in whether some forms of violence should be subject to legal sanctions or not (Tsawe and Mhele, 2022).

Intimate partner violence affects women's physical and mental health in many ways, and it can also have a negative impact on the child of those who have experienced it, as research indicates it goes as far as the death of the child at a young age (Kebede et al., 2022; Memiah et al., 2020). Thus, a history of violence might be a risk factor for a variety of illnesses and mental disorders. As research indicates there is a strong correlation between men's acceptance of wife-beating as a legitimate method of resolving marital conflicts and the actual occurrence of such behavior (Chirwa et al., 2018; Yoshikawa et al., 2014).

Previous studies indicated that those who accept the justification of IPV are mainly uneducated, unemployed, younger men, separated men, men from rural area, men with no exposure to media, men from the poorest wealth index group and those who believe IPV is a social norm in their communities (Darteh et al., 2021; Bukuluki et al., 2021; Schuler et al., 2012; Trott et al., 2017; Lawoko, 2008). Some studies in Africa tried to address the justification or attitude of intimate partner violence among women (Bukuluki et al., 2021; Trott et al., 2017; Doku and Asante, 2015; Husnu and Mertan, 2017; Atomssa et al., 2021). However, studies conducted to understand the justification or attitude of men toward IPV are rare (Oyediran, 2016; Pierotti, 2013). A study in sub-Saharan African countries using DHS data from 2010 to 2015 tried to show justification for IPV in men (Darteh et al., 2021).

Understanding the viewpoints, attitudes, or justifications of men on acts of intimate partner violence (IPV) against women is crucial for those working to prevent women from being subjected to this type of abuse and to create interventions that are culturally relevant. This study intends to expand upon earlier works and demonstrate how a variety of individual and community level factors associate with the justification of physical IPV among men in East Africa using the most recent DHS data (2015–2022).

## Method

### Source of data and study population

**Figure 1:**
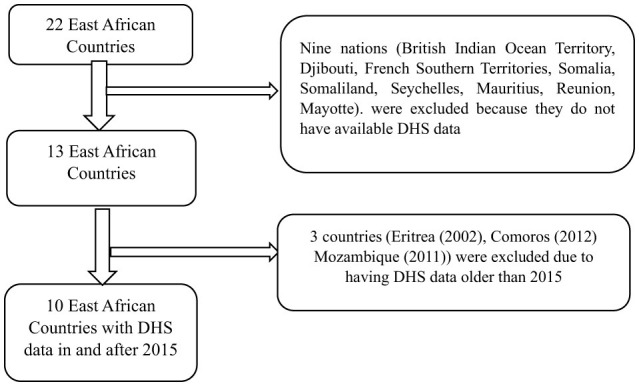
Country selection procedure.

The African continent has been divided into five regions by the UN Statistics Division with 22 nations (British Indian Ocean Territory, Burundi, Comoros, Djibouti, Ethiopia, Eritrea, French Southern Territories, Kenya, Madagascar, Malawi, Mauritius, Mozambique, Mayotte, Reunion, Rwanda, Seychelles, Somalia, Somaliland, Tanzania, Uganda, Zambia, and Zimbabwe) being part of the largest region East Africa (United Nations Statistics Division, 2024). From these 22 countries, nine of them (British Indian Ocean Territory, Djibouti, French Southern Territories, Somalia, Somaliland, Seychelles, Mauritius, Reunion, and Mayotte) do not have available DHS data. And from the remaining 13 countries that have available DHS data, three countries [Eritrea (2002), Comoros (2012), and Mozambique (2011)] were excluded due to having DHS data older than 2015 (Figure 1). So, in this study we included 10 countries having DHS data conducted in and after 2015 (Table 1).

**Table 1:** Country, survey year, and weighted sample size for the 10 East African countries.

**Country**	**Survey year**	**Weighted sample size**
Burundi	2016/17	8,755
Ethiopia	2015	7,471
Kenya	2022	13,915
Madagascar	2021	11,169
Malawi	2015/16	4,347
Rwanda	2019/20	6,961
Tanzania	2022	5,874
Uganda	2016	5,908
Zambia	2018	6,428
Zimbabwe	2015	8,656

Data from the male file (MR) of the most recent DHSs, which were carried out in 10 East-African countries, was used in this study. Demographic health surveys are national surveys that are gathered mostly from developing nations in Asia and Africa. Permission to utilize DHS data was obtained by providing a brief explanation of the study on the DHS official website https://dhsprogram.com/data/available-datasets.cfm

DHS interviews with men between the ages of 15 and 59 years. Choosing clusters or Enumeration areas (EAs) is the first step and then systematic sampling of the households enumerated in each cluster or EAs was done in the second stage. Interviews were conducted with all men aged 15 to 59 who were de facto. A weighted sample of 74,494 men who were either married or living with a partner were included in the study.

### Dependent variable

For our study, we consider justification of physical intimate partner violence (IPV) as an outcome variable, which was recoded as binary with 0 denoting no and 1 denoting yes and was generated from questions asked to men to understand their view about if some conditions could be justifiable to perform IPV. This was obtained from the participants' responses to the following items. “In your opinion, is a husband justified in hitting or beating his wife in the following situations?” (1) Wife goes out without telling him, (2) wife neglects the children, (3) wife argues with husband, (4) wife refuses to have sex with husband, and (5) wife burns food. The responses to questions for each of these responses were coded as “yes” and “no.” In our study, men who answered “yes” to at least one of the conditions in which a husband hits or beats the wife were considered to justify physical IPV, while those who answered “no” to all five conditions were considered not to justify physical IPV and by this the outcome variable was defined (Darteh et al., 2021; Adu et al., 2022; Aboagye et al., 2021).

### Independent variable

The individual-level independent variables included in this study are: age of respondent (15–24, 25–34, 35–44, 45–54, 55+), Respondents' educational status (no education, primary, secondary, and above), Respondents working status (working, not working), Media exposure (exposure measured by frequency of listening to radio or reading a newspaper or magazine) Respondents who answered “yes” to at least one of the three questions were considered to be exposed to media, while those who answered “no” to all were considered not exposed to media. Wealth status (poor, middle, rich), Religion (orthodox, catholic, protestant, Muslim, other.), Number of wives (one, more than one), Number of living children (none, one, more than one), sex of head of household (male or female), Age of household head in years (15–24, 25–34, 35–44, 45–54, 55+).

Community level independent variables included in our study are Place of residence (urban, rural), Community level education (low, high) constructed from respondents' educational status, Community level poverty (low, high), constructed from wealth index, Community level media exposure (low, high) constructed from individual level media exposure and Country Income (LMIC, LIC) classified based on World bank list of economies 2023–2024 (World Bank, 2023). Kenya, Tanzania, Zambia and Zimbabwe were classified as Lower middle-income countries and Burundi, Ethiopia, Madagascar, Malawi, Rwanda, and Uganda were classified as Lower income countries.

### Data analysis

Using STATA version 17, descriptive and inferential statistical analyses were carried out, and country-specific percentages are used to present descriptive data. To demonstrate the relationship between the independent and dependent variables, multilevel logistic regression was used, and variables with a *p*-value < 0.25 in the bi-variable analysis were candidates for the multivariable analysis. A *p*-value of 0.25 was chosen to reduce the risk of excluding variables that may become significant in the presence of other variables. In the multivariable analysis, an adjusted odds ratio with a 95% CI and a *p*-value < 0.05 was used to identify factors associated with the justification of IPV. We conducted a multilevel logistic regression analysis with both random and fixed effects. We created multilevel mixed-effects supplementary logistic regression models that could take into account the DHS's stratified multistage sampling method and assess the influence of hierarchical ordering (Primary sampling units and regions) on the variance of related factors (Amegbor and Pascoe, 2021). We assessed whether observations within the same country (or any hierarchical structure) are independent after accounting for the hierarchical structure by using intra-class correlation (ICC), media odds ratio (MOR), and likelihood ratio. We have also checked if the log odds of physical IPV are linearly related to the independent variables using the Box-Tidwell test (*p*-value > 5%). A variance inflation factor (VIF) has been used to test for multicollinearity among variables, and there was none (the VIF ranged from 1.2 to 5.49, with a mean of 2.66).

### Model building

A multilevel model was fitted due to the DHS data set hierarchical and clustering nature, since the prevalence of justification of IPV varies between clusters. In order to assess the presence of cluster variability, ICC was used, and also MOR and proportional change in variance (PCV) were computed to measure the variation of justification of IPV between clusters. Four models were fitted; the null model was without the independent variables, and the ICC in this model showed there is cluster variability (ICC = 16.9). Model 1 was fitted with the outcome variable and individual-level independent variables. Model 2 was fitted by the outcome variable and community-level factors. The last model, which is model 3, was fitted by the outcome variable and both individual and community-level factors. The model with the lowest Akaike information criterion (model 3) was selected as a best fitted model. ICC was calculated for each model by $ICC=\frac{VA}{VA+3.29}*100\%$ (Liyew and Teshale, 2020; Merlo et al., 2005; Asmamaw et al., 2023). PCV were calculated for each model, *PCV*= $\left(\frac{Vnull-Vm1,m2,m3}{Vnull}\right)*100\%$. Where Vnull = the variance in the Null model, Vm1 = variance in Model one, Vm2 = variance in Model two, Vm3 = variance in Model three. MOR was calculated for each model = *exp*(*0.95*√*VA*).

## Result

### Socio-demographic characteristics of respondents

Largest percentages of men (35%) were aged 25–34, with 16% of men from Kenya and 47% having completed primary school. Most men were currently employed (95%), had media exposure (83%), lived in male-headed households (95%), were monogamously married (94%), resided in rural areas (74%), and 56% were from lower-income countries. Additionally, 43% of men belonged to the rich wealth group (Table 2).

**Table 2:** Background characteristics of participants.

**Individual level variables**	**Frequency**	**Percentage**	**Community level variables**	**Frequency**	**Percentage**
**Men's age**			**Residence**		
15–24	6,186	7.78%	Urban	20,684	26.02%
25–34	28,094	35.35%	Rural	58,800	73.98%
35–44	26,159	32.91%	**Community level education**		
45–54	16,108	20.07%			
+55	2,936	3.69%			
**Men's educational status**	Low	39,744	50%		
	High	39,740	50%		
No education	12,022	15.12%	**Community level media exposure**		
Primary	37,118	46.70%	Low	40,974	51.55%
Secondary+	30,344	38.12%	High	38,510	48.45%
**Men's current working status**			**Community level poverty**		
Not working	3,821	4.81%	Low	40,154	50.52%
Working	75,663	95.19%	High	39,330	49.48%
**Wealth index**			**Country income**		
Poor	29,195	36.73%	LMIC	34,874	43.88%
Middle	15,761	19.83%	LIC	44,610	56.12%
Rich	34,527	43.44%			
**Media exposure**					
No	13,537	17.03%			
Yes	65,940	82.97%			
**Age of household head**					
15–24	4,938	6.21%			
25–34	26,297	33.08%			
35–44	25,919	32.61%			
45–54	16,511	20.77%			
+55	5,820	7.32%			
**Number of living children**					
None	4,668	5.87%			
One	11,667	14.68%			
More than one	63,149	79.45%			
**Number of wives**					
One	80,001	94.42%			
More than one	4,727	5.58%			
**Sex of household head**					
Male	75,413	94.88%			
Female	4,071	5.12%			
**Religion**					
Orthodox	43,151	30.19%			
Catholic	44,166	30.90%			
Protestant	15,764	11.03%			
Muslim	12,097	8.46%			
Others	27,764	19.42%			

### Pooled prevalence of justification of IPV in East African countries

**Figure 2:**
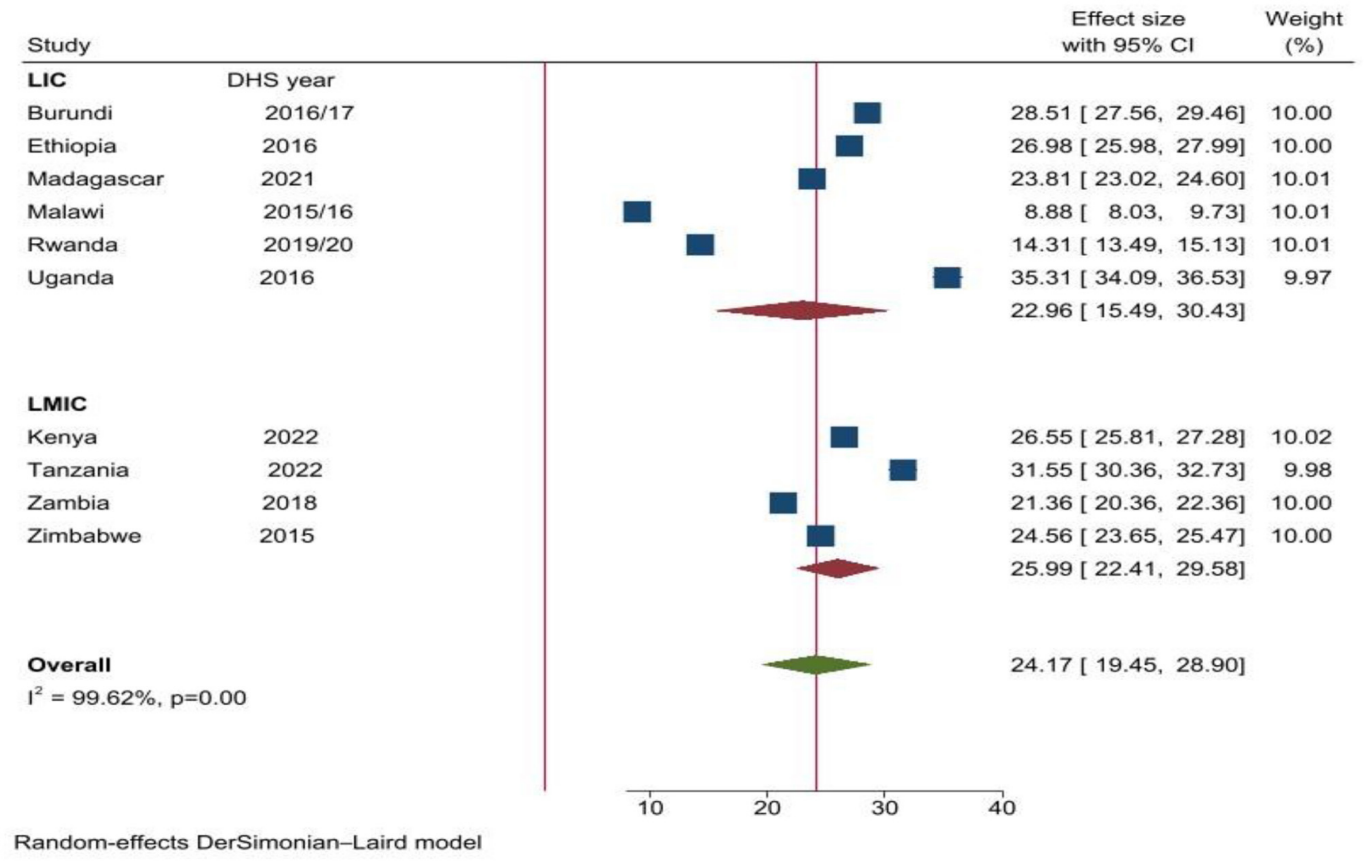
Forest plot of overall prevalence of justification of IPV among married men in East Africa countries from 2015 to 2022.

The pooled prevalence of justification of physical IPV among married men in East Africa was 24.17% (95% CI: 19.45–28.90). The highest prevalence was from Uganda 35.31% (95% CI: 34.09–36.53) and the lowest from Malawi 8.88% (95% CI: 8.03–9.73; Figure 2).

### Multilevel logistics regression

Table 3 shows the result of the multilevel analysis used to assess the determinants of justification of Physical IPV among men who are either married or living with a partner as if married in East Africa.

**Table 3:** Multilevel logistic regression analysis.

**Variables**	**Null model**	**Model 1 AOR (95%CI), *p*-value**	**Model 2 AOR (95%CI), *p*-value**	**Model 3 AOR (95%CI), *p*-value**
**Men's age**				
15–24		1	1	1
25–34		0.68 (0.62–0.75), **0.000**		0.67 (0.60–0.74), **0.000**
35–44		0.54 (0.49–0.61), **0.000**		0.53 (0.48–0.57), **0.000**
45–54		0.43 (0.38–0.49), **0.000**		0.43 (0.38–0.49), **0.000**
+55		0.34 (0.29–0.39), **0.000**		0.39 (0.34–0.45), **0.000**
**Men's educational status**				
No education		1		1
Primary		0.83 (0.79–0.87), **0.000**		0.80 (0.76–0.84), **0.000**
Secondary+		0.61 (0.58–0.65), **0.000**		0.55 (0.52–0.59), **0.000**
**Men's current working status**				
Not working		1		1
Working		1.08 (1.00–1.16), 0.048		1.19 (1.10–1.29), **0.000**
**Wealth status**				
Poor		1		1
Middel		0.86 (0.82–0.90), **0.000**		0.88 (0.84–0.92), **0.000**
Rich		0.70 (0.67–0.73), **0.000**		0.77 (0.73–0.80), **0.000**
**Media exposure**				
No		1		1
Yes		1.03 (0.95–1.05)		0.96 (0.92–1.01)
**Number of wives**				
No		1		1
More than one		1.31 (1.22–1.40), **0.000**		1.29 (1.21–1.39), **0.000**
**Sex of household head**				
Male		0.86 (0.82–0.95), **0.000**		0.90 (0.85–0.98), **0.021**
Female		1		1
**Number of living children**				
None		1		1
One		0.94 (0.86–1.01)		0.93 (0.86–1.00)
More than one		1.02 (0.93–1.08)		0.99 (0.92–1.07)
**Age of household head**				
15–24		1		1
25–34		1.25 (1.12–1.39), **0.000**		1.22 (1.10–1.36), **0.000**
35–44		1.21 (1.07–1.35), **0.001**		1.16 (1.03–1.30), **0.009**
45–54		1.28 (1.13–1.44), **0.000**		1.21 (1.07–1.36), **0.002**
+55		1.35 (1.20–1.52), **0.000**		1.22 (1.08–1.37), **0.001**
**Community level variables**				
**Residence**				
Urban			1	1
Rural			1.55 (1.48–1.62), **0.000**	1.15 (1.09–1.21), **0.000**
**Country income**				
LMIC			1	1
LIC			0.98 (0.91, 1.07)	1.04 (0.95, 1.14)
**Community level education**				
Low			1	1
High			0.78 (0.75–0.80), **0.000**	0.79 (0.66–0.72), **0.000**
**Community level media exposure**				
Low			1	1
High			0.96 (0.86–1.08)	0.98 (0.88–1.10)
**Community level poverty**				
Low			1	1
High			1.17 (1.06–1.30), **0.000**	1.11 (1.05–1.22), **0.035**
**Random effect**				
LLH	−45,919.3	−45,112	−45,591.1	−44,898.3
Variance	0.67	0.60	0.59	0.57
ICC	16.9%	15.1%	14.8%	14.3%
PCV	Reference	10.4	11.9	14.9
AIC	91,842.6	90,264	91,196.3	89,846.7
MOR	2.17	2.07	2.05	2.03

The odds of justifying physical IPV among husbands and partners with higher ages had 33% (AOR: 0.67 CI: 0.60–0.74), 46% (AOR: 0.54 CI: 0.48–0.58), 57% (AOR: 0.43 CI: 0.38–0.49), and 62% (AOR: 0.38 CI: 0.34–0.45) reduced chance of justifying IPV for the 25–34, 35–44, 45–54, and 55–64 age groups, respectively, compared to the younger age group (15–24). The odds of justifying physical IPV are also reduced by 20% (AOR: 0.80 CI: 0.76–0.84) and 23% (AOR: 0.77 CI: 0.73–0.80) for husbands and partners having primary education, secondary education, and a higher level of education, respectively, as compared to those with no education. Similarly, the household wealth index is also one of the significant determining factors of justifying physical IPV, and the odds of justifying physical IPV are reduced by 78% (AOR: 0.22 CI: 0.84–0.90) and 23% (AOR: 0.77 CI: 0.73–0.80) for men in the middle and rich wealth groups, respectively, when compared to men in the poor wealth group. Working status is also one factor associated with justifying physical IPV. The likelihood of justifying physical IPV is 1.19 (AOR: 1.19, CI: 1.10–1.29) times higher among husbands and partners who are working than those with no jobs. Likewise, the number of wives was also an associated factor for justifying physical IPV; the odds of justifying physical IPV violence are 1.29 (AOR: 1.29 CI: 1.21–1.39) times higher for men having more than one wife or partner compared to men having only one wife. When it comes to sex of the head of the household, men living in male-headed households had a 10% (AOR: 0.90 CI: 0.85–0.98) reduced chance of justifying physical IPV than those living in female-headed households. Results also show that men residing in households with older household heads are more likely to justify physical IPV. The odds of justifying physical IPV are 1.22 (AOR: 1.22 CI: 1.10–1.36), 1.16 (AOR: 1.16 CI: 1.03–1.30), 1.21 (AOR: 1.21 CI: 1.07–1.36), and 1.22 (AOR: 1.22 CI: 1.08–1.37) times higher for men living in households whose household heads were in the 25–34, 35–44, 45–54, and 55+ age groups, respectively, when compared to men living in younger household heads (15–24 years).

In addition to the above individual-level factors, the place of residence, community-level wealth, community-level education, and country-level income status are the community-level factors that determined the justification of physical IPV.

The odds of justifying physical IPV among rural respondents is 1.15 (AOR = 1.15; 95% CI: 1.09–1.21) times higher compared to respondents from urban areas, and the odds of justifying IPV is also 1.11 (AOR = 1.11; 95% CI: 1.05–1.22) times higher for husbands and partners who live in a higher poverty community when compared to those living in low poverty communities. The odds of justifying physical IPV is reduced by 21% (AOR = 0.79; 95% CI: 0.66–0.72), for respondents living in uneducated communities than those living in highly uneducated communities.

### Random effect

The ICC in the empty model shows that 16.9% of the variance in justifying physical intimate partner violence is a result of cluster differences. Moreover, the MOR value in the empty model showed that 2.17 times the odds of difference in justifying physical intimate partner violence are attributed to differences between clusters. The ICC showed a decrease as we moved from the empty model (ICC = 16.9) to the final model 3 (ICC = 14.3). The PCV in the final model explained that 14.9% of the variation in justifying physical intimate partner violence is a result of individual and community-related factors. Furthermore, the median odds ratio (MOR) in the final model was 2.03, if the respondent moved from a cluster with a low prevalence of justified IPV to a cluster with a high prevalence of justifying physical IPV, the median increase in the odds of justified IPV would increase by 2.03 times. Model three had a small Deviance (89,796.6) and AIC (89,846.7) and was chosen as the best-fitted model (Table 3).

## Discussion

The present study investigated the pooled prevalence and assessed individual and community- level factors associated with the justification of physical IPV among men in east Africa, a region where IPV is widespread and public justification is common (Tessema et al., 2023; Kebede et al., 2022; Darteh et al., 2021; Asmamaw et al., 2023). The pooled prevalence of justification of IPV among married men in East Africa in our study was 24.17% (95% CI: 19.45–28.90). This finding is lower than the study conducted on sub-Saharan countries between 2003 and 2007 and from a study conducted in India, which shows 33% of men justifying IPV (Darteh et al., 2021), also lower than a study conducted in India with 42% of men justified IPV (Pradhan and De, 2024). The prevalence of justifying physical IPV in East Africa is higher than the study conducted in Ghana (Ola, 2022) and Ethiopia (Abeya, 2015), 12.4 and 7% respectively. The possible explanation for this difference might be that since the prevalence in our study was pooled of 10 east African countries, and these other studies were conducted on a single country. The other reason for the observed difference in the prevalence could be different year of the study period. Furthermore, cultural norms and traditions on the issue of IPV such as bride prices, dowries, or planned weddings, patriarchal beliefs, and gender-inequitable attitudes, might be different from population to population (Sikweyiya et al., 2020; Shakya et al., 2022). Studies show that attitudes toward IPV, gender inequity, and traditional gender roles vary between cultures. Despite the patriarchal nature of many African countries (Uthman et al., 2009), there are significant cultural differences in how much male superiority and female inferiority are regarded and ingrained in everyday life (Zark and Satyen, 2022).

Our finding showed that older men are less likely to justify IPV than younger men. Our result is similar to previously conducted studies (Darteh et al., 2021; Trott et al., 2017; Okenwa-Emegwa et al., 2016; Waltermaurer, 2012). To explain, the first way to look at it is from the perspective of social learning theory, which contends that young people accept physical abuse of women as a form of discipline for misbehavior (Akers and Jennings, 2015). For example, children who grow up in families where intimate partner violence (IPV) is common may learn from their dads how to justify IPV and eventually become inclined to rationalize any episode of IPV (Singh et al., 2012; Jones, 2021). This tendency is undoubtedly going to produce a more positive attitude toward IPV, but it will also likely result in a decrease when they learn more about the impacts of IPV and obtain more trustworthy information from their environment (Abeya, 2015). Other factors, such as drug abuse and peer-pressure might also explain this finding, this could be explained by, in order to be accepted by their peers' young males may encounter pressure to live up to hyper-masculine norms, which might involve controlling others with violence, suppressing emotions, and dominating partners. Moreover, a person's self-control, impulsivity, and inhibitions can all be negatively impacted by substance use which will increase likelihood of aggressive conduct then leading to justifying IPV (Mulawa et al., 2018; McKool et al., 2021).

In terms of educational status, men with primary, secondary and above educational status, are less likely to justify IPV than men with no educational status and this result is in line with previously conducted studies (Darteh et al., 2021; Lawoko, 2008; Abeya, 2015; Taylor et al., 2017; Ola, 2022; Serrano-Montilla et al., 2020). A possible justification for this might be that men who are uneducated may have the perception of viewing their wife or partner as their own property (Burazeri et al., 2005; Sayem et al., 2012). Being uneducated, they may cling to harmful cultural ideas and have fewer opportunities to learn about rights to safety and international norms regarding gender equity, which gives them the right to control and abuse (Antai, 2011). Moreover, the findings from this study emphasizes the importance of men's education in reducing the justification of IPV. In addition to providing opportunities for self-discovery, reflection on human rights, personal development, and constructive contributions to society, higher education may also contribute to the rise of contemporary values, equality, self-respect, respect for others, and a decrease in violent tendencies (Ekawu, 2024). Despite this, a study in Nigeria showed the attitude toward IPV was higher for men having primary, secondary and above educational status, reporting a result different from our study (Okenwa-Emegwa et al., 2016).

Our study's findings are consistent with earlier studies, that men who are employed are more likely than those who are not to justify intimate partner violence (Darteh et al., 2021; Gennari et al., 2017). In some parts of the Africa, there may be cultural expectations about men's responsibilities as family providers and wage earner (Shakya et al., 2022). Working men may experience pressure to uphold these traditional gender norms, which may include controlling their partners and defending IPV as a tactic for dominance. Another possible explanation might be that men who are working may believe they are in a better financial position in a relationship and feel more independent, which could cause them to respond angrily and defend beating their wives (Gennari et al., 2017). So, in an attempt to maintain dominance or authority, controlling behaviors like IPV may be justified due to this perceived imbalance in power (Scott-Storey et al., 2022). However, since ~95% of respondents were employed, indicating a large work status imbalance in the sample. This lack of variation could affect how our finding about working status and attitudes against IPV are interpreted.

In the case of wealth status, men from the poor wealth index had a high chance of justifying IPV compared to men from middle and rich wealth index; similar findings were reported in previous studies as well (Darteh et al., 2021; Abeya, 2015; Clare et al., 2021). The possible explanation for this may be that financial instability and poverty can increase relationship stressors and raise the possibility of violence, according to studies, financial difficulties may increase relationship stress since they can cause frustration, despair, and a breakdown in communication. In these circumstances, IPV may be viewed as a means of reclaiming control as well as a response to conflict (Mulia, 2008; Jewkes, 2002).

In addition, those from low-income families, might also be more inclined to agree that physical IPV is justified because of their personal exposure, that is experiencing violence either directly or indirectly, such as seeing intimate partner violence (IPV) in the home, growing up in communities that encourages such violence, or even being victims of IPV themselves due to this their opinions may be influenced and leading them to justify IPV (Mitchell et al., 2013; Chowdhury and Mathur, 2021; Copp et al., 2019). On the contrary, other previous studies indicated that men from the poor wealth households had a better view of IPV than men from middle and rich wealth households (Tran et al., 2016; Taylor et al., 2017; Adu, 2023).

Men who are polygamous are more likely to justify intimate partner violence compared to those with only one wife and this result has also been noted in earlier research (Oyediran, 2016; Ola, 2022). Possible explanation for this might be, since polygyny is a patriarchal system where men are customarily granted more sexual rights than women, this practice may lead to justifying the physical form of IPV (Bowan, 2013). Since in most African countries, it is cultural and traditional right for men to marry more than one because of this unequal sexual privilege, polygamous men may believe they are entitled to greater rights than women, which may lead them to justify beating their wives (Amo-Adjei and Tuoyire, 2016).

Our study indicated that “sex of the household head” and “age of the household head” are factors significantly associated with the justification of physical IPV, even though these variables had not been mentioned as factors in previously conducted researches (Darteh et al., 2021; Oyediran, 2016; Pierotti, 2013). A study in Bangladesh shows that there is a relationship between intimate partner violence and the sex of the household head (Afiaz et al., 2020). The finding from our study showed that men that are living in households where males are head of household are less likely than those men who are living in a household where females are head of the household to justify IPV. This finding demonstrates that any household can experience IPV, regardless of who is considered the “head.” One possible explanation is that these families may strongly believe that violence is unacceptable in their home, prioritizing personal values and beliefs that emphasize respect and healthy relationships over violence (Barth and Jiranek, 2023). These values and beliefs might have been instilled through education, upbringing, religious instruction, or in some cases firsthand experiences (Abeya, 2015; Waltermaurer, 2012). Moreover, some homes may have a male head who exemplifies respectful behavior toward spouses and non-violent conflict resolution. So individuals living in these kind of households may reject violence as appropriate behavior in relationships (Asghar et al., 2017). Furthermore, it's also important to recognize that not all households with a male head are the same. Male domination may still encourage patriarchal views in some situations, and other elements like education, social mores, or exposure to violence may be more important than home headship alone. However, since 95% of respondents were from households headed by men, indicating a significant bias in the sample. Therefore, the interpretation of our results regarding household power dynamics and the justification of IPV may be impacted by this low proportion of female-headed households.

The other finding is that men living with older household head have a higher chance of justifying physical intimate partner violence. A possible way to explain this may be traditions held by older household heads may have an impact on men's attitudes regarding intimate partner violence in those households since older heads of households, for instance, might be more inclined to support male-controlled viewpoints that defend or tolerate IPV as a form of correction or discipline. Men living in these households are likely to justify IPV. This finding is supported by previously conducted studies (Sayem et al., 2012; Saud et al., 2021).

Compared to men who live in urban areas, those who live in rural areas are more likely to justify physical IPV, Previous studies support this result as well (Darteh et al., 2021; Pradhan and De, 2024; Ola, 2022; Abeya, 2015). This can be explained by the wide spread of gender equality and other norms within urban households, given that men living in urban areas are more exposed to modern cultures which help to reduce IPV, by promoting values and norms that emphasize gender equality, individual rights, and non-violent conflict resolution (Jewkes, 2013). Moreover, compared to men in rural regions, urban men with greater levels of education are less likely to defend IPV, since education frequently promotes a heightened consciousness of human rights, gender equality, and the legal ramifications of intimate partner violence (IPV), which may result in a rejection of excuses for such behavior (Dalal, 2011; Ackerson et al., 2008; Boyle et al., 2009). Furthermore, men from communities that are highly impoverished and have low levels of education are more likely to defend IPV. This can be attributed to the persistence of harmful traditions, practices, and norms in these communities, such as the domination of wives, which are often passed down through generations (Bhushan and Singh, 2014; Benebo et al., 2018).

### Strengths and limitations of the study

The primary strength of the study was the use of nationally representative data (DHS) from the most recent 10 East African countries which could possess sufficient statistical power to identify the relationship between certain factors and the justification of physical IPV. Secondly the use of advanced statistical method a multilevel analysis to take into the data's hierarchical structure was another area of strength. Although this study has an important contribution to the study of justification for physical IPV, some limitations also exist, like the fact that DHS data set did not fully include some of the key variables that could have been linked to the justification of IPV, such as childhood exposure to IPV and experience of child abuse. Also, due to the cross-sectional nature of the data, we are unable to determine the temporal relationship between the independent and dependent variables. Since the DHS survey relied on respondents' self-reports, there may have been issues with recall bias during data collection procedure.

## Conclusion

This study found that the justification of physical intimate partner violence among men who are married or living with a partner in East Africa was related to both individual and community level factors. Even though its pooled prevalence is lower, there is a big difference between countries. Factors such as educational status, working status, age, number of wives, sex of head of household, age of the household head, residence, community level education, and community level poverty were the factors significantly associated with the justification of physical IPV. Policy makers and programs should advance men's educational standing, men's economic status, and increased media awareness. Governmental institutions and agencies, and non-government organizations should advocate against the justification of physical IPV against women through education and awareness campaigns and empowerment programs at the community level.

## Data Availability

Publicly available datasets were analyzed in this study. The datasets used for the study are publicly available from the DHS official website https://dhsprogram.com/data/available-datasets.cfm.
